# TGF-β1 induces senescence of bone marrow mesenchymal stem cells via increase of mitochondrial ROS production

**DOI:** 10.1186/1471-213X-14-21

**Published:** 2014-05-18

**Authors:** Junfang Wu, Jie Niu, Xiaopeng Li, Xianwei Wang, Zhikun Guo, Fenxi Zhang

**Affiliations:** 1 Key Laboratory of Henan Province for Medical Tissue Regeneration, Xinxiang Medical University, Xinxiang, Henan 453003, China; 2 The Third Affiliated Hospital of Xinxiang Medical University, Xinxiang 453003, China; 3 Department of Anatomy, Xinxiang Medical University, Xinxiang 453003, China

**Keywords:** Transforming growth factor beta 1, Bone marrow mesenchymal stem cells, Cell senescence, Senescence-associated-galactosidase activity, Mitochondrial reactive oxygen species

## Abstract

**Background:**

Bone marrow derived mesenchymal stem cells (bmMSCs) are multipotent cells that can differentiate into diverse cell types, including cardiomyocytes. BmMSC-based transplantation is capable of repairing acute and chronic myocardial infarction. Prior to the transplantation, MSCs are usually induced *in vitro* by biological reagents and chemicals for directional differentiation. Transforming growth factor beta (TGF-β) is one of the most commonly used biological reagents for induction of cardiomyocyte differentiation of bmMSCs. Previous studies have shown that TGF-β induces senescence in several cell types. However, whether TGF-β affects senescence of bmMSCs has not been elucidated. The goal of this study was to investigate the effect of TGF-β1 on senescence of bmMSCs and the underlying mechanisms.

**Results:**

We found that TGF-β1 increased activity of senescence-associated-galactosidase (SA-Gal) and production of mitochondrial reactive oxygen species (mtROS) in bmMSCs in a dose-dependent manner. TGF-β1 also significantly decreased expression of superoxide dismutase 2 (SOD2) and Id1, and increased expression of 4-Hydroxynonenal (4-HNE) subunits and p16 in bmMSCs in a dose-dependent manner. Pre-treatment with mtROS inhibitor acetyl-L-carnitine (ALCAR, 0.1 mM) significantly inhibited TGF-β1-induced mtROS production and SA-Gal activity.

**Conclusion:**

TGF-β1 can induce senescence of bmMSCs, which at least partially depends on mtROS production.

## Background

Mesenchymal stem cells (MSCs) are multipotent adult stem cells with a high capacity for self-renewal and capable of differentiating into a variety of cell types, including adipocytes, osteoblasts, chondrocytes, endothelial cells, cardiomyocytes and neurons [1,2]. Currently, MSCs have been widely used in regenerative medicine [3]. The most common source of MSCs is bone marrow MSCs (bmMSCs) [4]. Previous studies have shown that bmMSC transplantation has the potential to reduce infarct size and improve cardiac function in animal models of heart failure [5]. BmMSCs are usually induced *in vitro* with special reagents for directional differentiation before transplantation. Transforming growth factor beta (TGF-β) is one of the most commonly used biological reagents for inducing cardiomyocyte differentiation of MSCs [6-8].

Senescence would result in a permanent cell cycle arrest and make MSCs lose their self-renewal potential [9]. The high proliferative capacity and regenerative potential are main phenotypes of MSCs [10]. Loss of regenerative potential would limit their application in transplantation medicine. TGF-β1 has been demonstrated to induce senescence in tumor cells and other cell lines [11-13]. TGF-β1 has also been shown to increase production of mitochondrial reactive oxygen species (mtROS) in some cell lineages [14]. MtROS production involves aging and cell senescence [14-16]. However, whether TGF-β affects senescence of bmMSCs has still not been elucidated. The purpose of this study was to investigate the effect of TGF-β1 on senescence of bmMSCs and its relation to mtROS generation.

## Methods

### Materials and reagents

Recombinant mouse TGF-β1 and Senescence β-Galactosidase Staining Kit were purchased from Cell Signaling Technology (Danvers, MA, USA). MitoSOX™ Red superoxide indicator, L-glutamine, ProlongH Gold antifade reagent with DAPI and DMEM were purchased from Invitrogen Life Technologies (Carlsbad, CA, USA). Acetyl-L-carnitine, β-glycerophosphate and Oil Red O were purchased from Sigma-Aldrich (St. Louis, MO, USA). DAB Substrate Kit, PE-conjugated CD44 antibody, FITC-conjugated CD90 antibody, 4-HNE, SOD2, β-actin primary antibodies and HRP-conjugated secondary antibodies were purchased from Abcam (Cambridge, MA, USA). Alkaline phosphatase antibody was purchased from Santa Cruz Biotechnology (Santa Cruz, CA, USA). HyClone Fetal Bovine Serum (FBS) was purchased from Thermo Fisher Scientific Inc. (Cleveland, OH, USA). ECL Western-blotting substrate was purchased from Thermo Fisher Scientific (Rockford, IL, USA). The PVDF membrane was purchased from GE healthcare (Pittssburgh, PA, USA).

### Culture of bmMSCs

BmMSCs were isolated and cultured as recently published protocols [17,18]. In brief, bone marrow was harvested from tibia and femoral of C57BL/6 mice and cultured in DMEM supplemented with 15% FBS, 2 mM L-glutamine, 100 μg penicillin, and 100 μg streptomycin. After 3 hours incubation, the non-adherent cells were removed and the medium was replaced. A purified population of bmMSCs can be obtained following 3 weeks of culture. The 3^rd^ passage bmMSCs were used in the experiments. The animal use and study protocols were approved by the Ethics Committee of Xinxiang Medical University.

### Treatments of bmMSCs

BmMSCs were plated in 6-well or 12-well plates and treated with 1, 5 and 10 ng/mL recombinant mouse TGF-β1 for 24 hours. The cells cultured in the common medium served as control. In the subsequent experiments, cells were pretreated with 0.1 mM acetyl-L-carnitine (ALCAR) for 30 min, and then exposed to 5 ng/mL TGF-β1 for 24 hours.

### Osteogenic differentiation culture

The osteogenic differentiation culture was performed as previously reported protocols [19]. In brief, bmMSCs were plated in 24-well plates with round coverslips and cultured with DMEM supplemented with 2% FBS, 5 mM β-glycerophosphate and 50 μM L-ascorbic acid-2-phosphate for 3 weeks. The medium was changed every three days. The osteogenic differentiation was analyzed by immunochemistry staining to measure expression of alkaline phosphatase (ALP) which is a maker of osteoblasts. The immunochemistry staining was performed as standard protocols.

### Adipogenic differentiation culture

BmMSCs were plated in 24-well plates with round coverslips and cultured in the induction medium (DMEM supplemented with 10% FBS, 1 μM dexamethasone, 60 μM indomethacin, 10 μg/mL insulin, and 0.5 mM 3-isobutyl-1-methylxanthine) for 3 days. Subsequently, the cells were cultured in the maintenance medium (DMEM supplemented with 10 μg/mL insulin) for 3 weeks. The maintenance medium was changed every other day. The adipogenic differentiation was analyzed by Oil Red O staining.

### Immunofluorescence staining

BmMSCs were plated in 24-well plates with 10 mm round coverslips. After 24-hour culture, the cells were fixed with 4% buffered formaldehyde for 15 min and treated with 0.1% Triton X-100 for 10 min at room temperature. And then, the cells were incubated with 1% BSA/10% goat serum for 30 min, and subsequently incubated with PE-conjugated goat anti-mouse CD44 antibody for 1 hour at room temperature in the dark. After washing thrice with PBS, the cells were incubated with FITC-conjugated goat anti-mouse CD90 antibody for 1 hour at room temperature in the dark. After washing with PBS and deionized water, the cells were mounted on slides using ProlongH Gold antifade reagent with 4’,6-diamidino-2-phenylindole (DAPI), and imaged with a fluorescence microscope.

### Senescence-β-Galactosidase Staining

In this study, cell senescence was analyzed using a Senescence-β-Galactosidase Staining kit. BmMSCs were plated in 12-well plates. Following 24 hours of culture, the cells were treated with TGF-β1 for additional 24 hours. After treatments with TGF-β1, the cells were washed twice with PBS and fixed with 0.5 mL 1Х fixative solution for 15 min at room temperature. After rinsing twice with PBS, the cells were incubated with 1 mL β-Galactosdase staining solution in a dry incubator without CO_2_ at 37°C overnight. The cells were imaged with a microscope when β-Galactosdase staining solution is still on the plates. The blue-dye-positive cells were viewed as senescent cells.

### Mitochondrial ROS (mtROS) measurement

In this study, mtROS were measured using a MitoSOX™ Red mitochondrial superoxide indicator, as the manufacturer’s instructions. Briefly, bmMSCs were cultured in 24-well plates. After treatments with TGF-β1, the cells were incubated with 5 μM MitoSOX™ reagent working solution for 10 min at 37°C in the dark. After washing thrice with warm PBS, the fluorescence was imaged with a fluorescent microscope.

### Western-blotting assay

Proteins were extracted from the treated bmMSCs and separated by 12% SDS-Polyacrylamide gel electrophoresis (SDS-PAGE). Following electrophoresis, proteins were transferred to PVDF membranes. The membranes were blocked with 5% BSA in Tris-buffered saline with Tween-20 (TBS-T), and then incubated with rabbit anti-mouse 4-HNE, P16, Id1 and β-actin primary antibodies at 4°C overnight. Then, the blots were washed thrice with TBS-T, and subsequently incubated with HRP-conjugated secondary antibody for 1 hour at room temperature. The immunoreactive bands were visualized with enhanced chemiluminescence.

### Statistical analysis

Statistical analysis was performed with SPSS11.5 software. Data were presented as mean ± SD from 4 independent experiments. Univariate comparisons of means were evaluated using the Student *t* test and/or one-way ANOVA with Tukey’s post-hoc adjustment for multiple comparisons when appropriate. P < 0.05 was considered a statistically significant difference.

## Results

### Identification of bmMSCs

**Figure 1:**
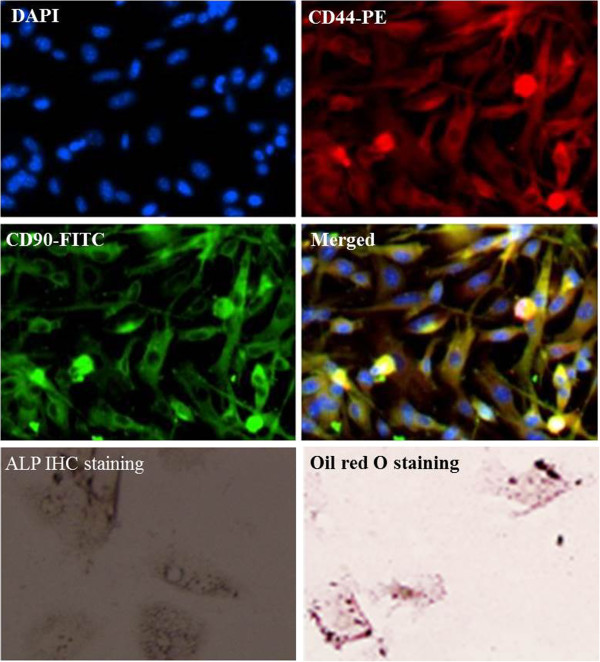
**Identification of bone marrow mesenchymal stem cells (bmMSCs).** Immunofluorescence assay shows that bmMSCs positively express MSC markers CD44 and CD90. Immunochemistry staining shows that alkaline phosphatase (ALP) is positively expressed in bmMSCs following 3 weeks of osteogenic differntiation culture. Oil Red O staining displays that lipid droplets are accumulated in a part of bmMSCs following 3 weeks of adipogenic differentiation culture.

Immunofluorescence staining showed that bmMSCs were positive for CD44 and CD90, which are MSC specific markers [1,20]. It is known that bmMSCs have the potential to differentiate to osteoblasts and apidocytes. High alkaline phosphatase (ALP) activity is an indication of successful differentiation of MSCs to osteoblasts [19]. Immunochemistry staining showed that most of bmMSCs positively expressed ALP after exposure to osteogenic differentiation medium for 3 weeks (Figure 1). Intracellular lipid vesicles are typically observed in adipocytes, which can be stained with Oil Red O. Our data showed that lipid droplets were accumulated in a part of bmMSCs after exposure to adipogenic differentiation medium for 3 weeks (Figure 1).

### TGF-β1 induces senescence of bmMSCs

**Figure 2:**
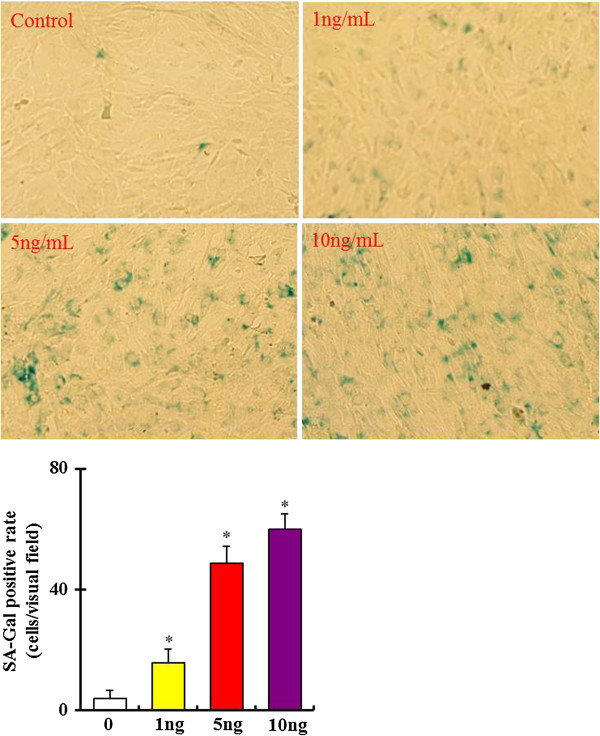
**Dose**–**response of β**-**galactosidase activity in bone marrow mesenchymal stem cells (bmMSCs) after exposure to 0, 1, 5 and 10 ng/mL recombinant mouse transforming growth factor β1 (TGF**-**β1) for 24 hours.** Bar graphs represent mean ± SD (4 independent experiments/group). *P < 0.05 vs. control.

**Figure 3:**
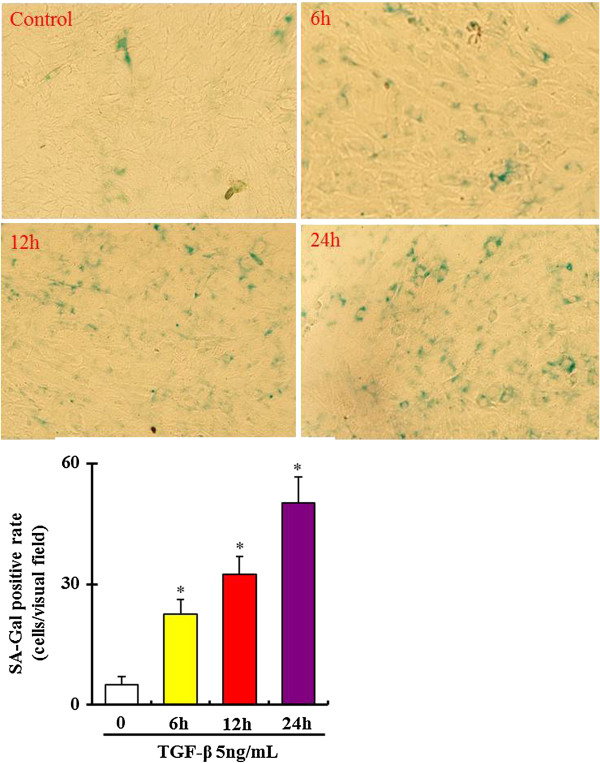
**Time**-**response of β**-**galactosidase activity in bone marrow mesenchymal stem cells (bmMSCs) after exposure to 5 ng/mL recombinant mouse transforming growth factor β1 (TGF-β1) for 0**–**24 hours.** Bar graphs represent mean ± SD (4 independent experiments/group). *P < 0.05 vs. control.

β-Galactosidase activity at PH 6 is present only in senescent cells and viewed as a special marker for cellular senescence [21,22]. In this study, the senescence of bmMSCs was analyzed using a Senescence-β-Galactosidase Staining kit. As shown in Figure 2, senescence-associated-galactosidase (SA-Gal) activity was significantly increased in bmMSCs in a dose-dependent manner after exposure to 1, 5 and 10 ng/mL TGF-β1 for 24 hours. SA-Gal activity was also increased in bmMSCs in a time-dependent manner as the cells were exposed to 5 ng/mL TGF-β1 (Figure 3).

### Expression of aging markers in bmMSCs after exposure to TGF-β1

**Figure 4:**
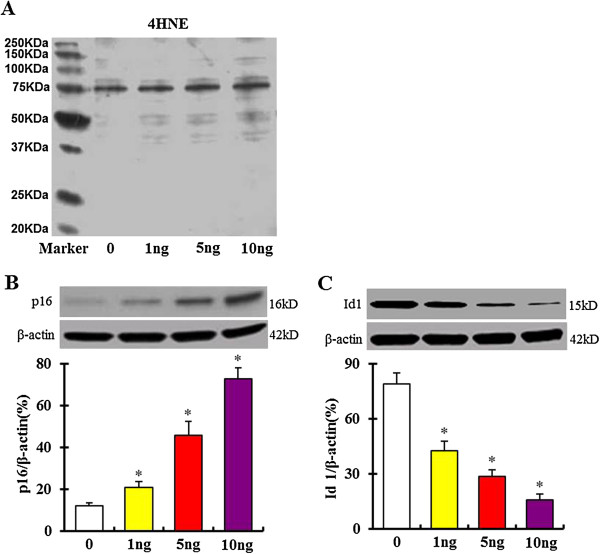
**Western-blot assay shows expression of aging marker proteins 4-Hydroxynonenal (4**-**HNE), p16 and Id1 in bmMSCs after exposure to 0, 1, 5 and 10 ng/mL TGF-β1 for 24 hours. A**. 4-HNE expression; **B**. P16 expression; **C**. Id1 expression. Bar graphs represent mean ± SD (4 independent experiments/group). *P < 0.05 vs. control.

4-Hydroxynonenal (4-HNE) is a highly reactive aldehyde generated by the exposure of polyunsaturated fatty acids to peroxides and ROS. 4-HNE plays a key role in signal transduction and numerous cell cycle events. The expression of 4-HNE subunits has also been involved in the senescence-associated ROS production and viewed as a marker of aging [23-25]. Our result showed that expression of 4-HNE subunits was markedly increased in bmMSCs as the cells were exposed to 1, 5 and 10 ng/mL TGF-β1 for 24 hours (Figure 4A). P16 and Id1 are also important markers of aging [13,26]. Our western blot data showed that p16 expression was markedly increased, but Id1 expression was decreased in bmMSCs as the cells were exposed to 1, 5 and 10 ng/mL TGF-β1 for 24 hours (Figure 4B and C).

### TGF-β1 increases mtROS production in bmMSCs

**Figure 5:**
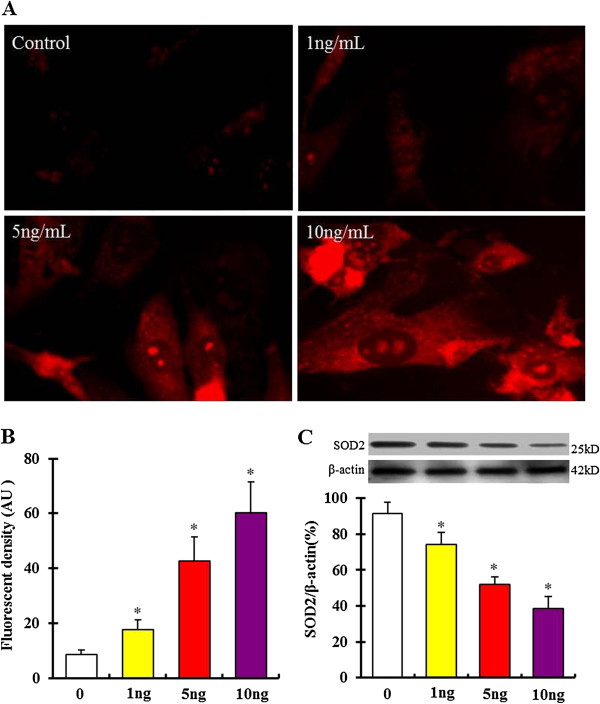
**Mitochondrial reactive oxygen species (mtROS) production in bone marrow mesenchymal stem cells (bmMSCs) after exposure to 0, 1, 5 and 10 ng/mL TGF-β1 for 24 hours. A**. MitoSOX™ Red Indicator staining shows mtROS in bmMSCs after exposure to 0, 1, 5 and 10 ng/mL TGF-β1 for 24 hours. **B**. Quantification of fluorescence density of mtROS. **C**. Western blot assay shows expression of superoxide dismutase 2 (SOD2) in bmMSCs after exposure to 0, 1, 5 and 10 ng/mL TGF-β1 for 24 hours. Bar graphs represent mean ± SD (4 independent experiments/group). *P < 0.05 vs. control.

It is known that mtROS involve cellular aging [15,27]. In this study, mtROS were measured using a MitoSOX Red indicator kit. As shown in Figure 5A and B, mtROS were markedly increased in bmMSCs after exposure to 1, 5 and 10 ng/mL TGF-β1 for 24 hours. Superoxide dismutase 2 (SOD2) is a mitochondrial matrix enzyme that protects mitochondria against oxidative radicals [16]. Previous studies have shown that deletion and downregulation of SOD2 both cause cellular senescence through increasing mtROS production [16,28]. Our results showed that SOD2 expression was significantly decreased in bmMSCs after exposure to 1, 5 and 10 ng/mL TGF-β1 for 24 hours (Figure 5C).

### ALCAR inhibits TGF-β1-induced mtROS production and bmMSC senescence

**Figure 6:**
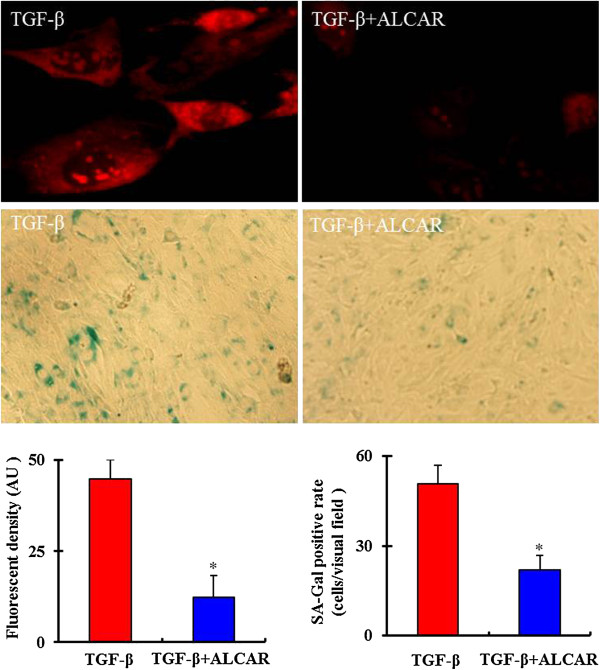
**Mitochodrial ROS (mtROS) specific inhibitor acetyl-L-carnitine (ALCAR) inhibits TGF-β1-induced mtROS generation and bmMSC senescence.** Bar graphs represent mean ± SD (4 independent experiments/group). *P < 0.05 vs. treatments with 5 ng/mL TGF-β1.

**Figure 7:**
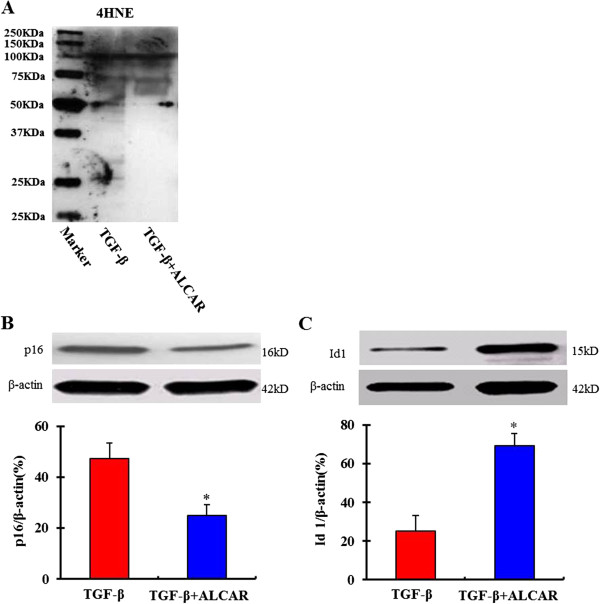
**Expression of aging markers 4-Hydroxynonenal (4-HNE), p16 and Id1 in bmMSCs as the cells were exposed to acetyl-L-carnitine (ALCAR, 0.1 mM) and TGF-β1 (5 ng/mL) simultaneously. A**. 4-HNE expression; **B**. P16 expression; **C**. Id1 expression. Bar graphs represent mean ± SD (4 independent experiments/group). *P < 0.05 vs. treatments with 5 ng/mL TGF-β1.

Acetyl-L-carnitine (ALCAR) is a specific inhibitor of mtROS [29,30]. Our results showed that ALCAR (0.1 mM) significantly inhibited TGF-β1-induced mtROS generation and SA-Gal activity in bmMSCs (Figure 6). In addition, ALCAR also markedly inhibited TGF-β1-induced 4-HNE subunits expression, and promoted Id1 expression (Figure 7).

## Discussion

In the present study, we investigated the effect of TGF-β1 on senescence of bmMSCs. Our results showed that treatments with TGF-β1 (1 ~ 10 ng/mL) increased SA-Gal activity and mtROS production in bmMSCs in a dose dependent manner. The aging promoter p16 and oxidative stress inducer 4-HNE were markedly increased; however, their opponents Id1 and SOD2 were significantly decreased in bmMSCs after exposure to TGF-β1. Application of mtROS inhibitor acetyl-L-carnitine significantly inhibited TGF-β1-induced mtROS production and bmMSC senescence. These findings demonstrate that TGF-β1 can cause senescence of bmMSCs, which involves mtROS production.

BmMSCs are the most promising sources of stem cells and have been widely applied to treat cardiac diseases [5]. Transplantation of bmMSCs has the potential to reduce infarct size and improve ventricular compliance after myocardial infarction [5]. BmMSCs are usually pre-treated with some biological reagents and chemicals, such as 5-azacytidine, TGF-β, angiotensin II, hypoxia-inducible factor-1 alpha (HIF-1α) and slingshot-1 L (SSH1L) for cardiomyocyte differentiation, prior to transplantation [6,7,31-33]. Among these reagents, TGF-β is the most commonly used one. Previous studies have shown that TGF-β1 and -β2 have the potential to induce senescence of tumor cells and other kinds of normal tissue cells [11-13]. Our results showed that treatments with TGF-β1 markedly increased SA-Gal activity in bmMSCs, which showed that these cells were undergoing senescence. Senescence would lead to phenotype changes and low proliferation of bmMSCs, which reduces the efficiency of bmMSC-based transplantation [34].

Our results showed that TGF-β1 induced expression of 4-HNE subunits. 4-HNE, a major lipid peroxidation (LPO) product, plays key roles in signal transduction pathways, and participates in cell cycle events. While lower levels of intracellular 4-HNE are beneficial to cells, possibly promoting cellular proliferation; however, higher levels cause toxic responses in cells, including cell senescence and apoptosis. Expression of 4-HNE has been proved to induce cell senescence and organ aging [23,35,36]. We found that p16, an important regulator of aging, was markedly upregulated in the TGF-β1-treated bmMSCs; however, Id1, a negative regulator of p16, was markedly downregulated in these cells. These data were consistent with previous reports from other groups, which showed that p16 protein was highly expressed, but Id1 protein was downregulated in the senescent cells and aged tissues [13,26,35].

More interestingly, mtROS production was also markedly increased in bmMSCs after exposure to TGF-β1. This data is also consistent with expression of 4-HNE that has been widely accepted as an inducer of cellular oxidative stress [23,25]. Previous studies have shown that TGF-β1 can increase mtROS production in tumor cells [14]. MtROS has also been known as key inducer of aging [37]. We also observed that SOD2 was significantly downregulated in bmMSCs after exposure to TGF-β1. SOD2 is known to be a key enzyme that protects mitochondria from ROS insult [16,38].

To further elucidate the role of mtROS in TGF-β1-induced bmMSC senescence, we treated bmMSCs with mtROS specific inhibitor acetyl-L-carnitine (ALCAR) when the cells were exposed to 5 ng/mL TGF-β1. Our results showed that ALCAR (0.1 mM) significantly inhibited TGF-β1-induced mtROS production and increase of SA-Gal activity. These data show that TGF-β1-induced senescence of bmMSCs at least partially depends on mtROS production.

## Conclusions

This study shows that TGF-β1, one of the most commonly used reagents for inducing cardiac differentiation of MSCs, causes senescence of bmMSCs. The action of TGF-β1 on bmMSC senescence depends on mtROS production, because blockade of mtROS production markedly inhibits TGF-β1-induced senescence of bmMSCs.

## Competing interests

The authors declared that they have no competing interests.

## Author’s contribution

WJ and NJ carried out cell culture and immunofluorescence, cellular chemistry, β-Galactosidase staining. WJ, LX and WX carried out ROS staining and Western-blotting assays. ZF designed the study, analyzed the data and wrote the manuscript. GZ helped ZF to design the study and participated in manuscript writing. All authors had read and approved the final manuscript.

## References

[B1] Zhang F Hong Y Liang W Ren T Jing S Lin J Co-culture with Sertoli cells promotes proliferation and migration of umbilical cord mesenchymal stem cells Biochem Biophys Res Commun 2012 427 86 90 10.1016/j.bbrc.2012.09.007 22975347

[B2] Ferroni L Gardin C Tocco I Epis R Casadei A Vindigni V Mucci G Zavan B Potential for neural differentiation of mesenchymal stem cells Adv Biochem Eng Biot 2013 129 89 115 10.1007/10_2012_152 22899379

[B3] Patel DM Shah J Srivastava AS Therapeutic potential of mesenchymal stem cells in regenerative medicine Stem Cells Int 2013 2013 15 10.1155/2013/496218 PMC3615627 23577036

[B4] Malgieri A Kantzari E Patrizi MP Gambardella S Bone marrow and umbilical cord blood human mesenchymal stem cells: state of the art Int J Clin Exp Med 2010 3 248 269 21072260 PMC2971538

[B5] Williams AR Hatzistergos KE Addicott B McCall F Carvalho D Suncion V Morales AR Da Silva J Sussman MA Heldman AW Hare JM Enhanced effect of combining human cardiac stem cells and bone marrow mesenchymal stem cells to reduce infarct size and to restore cardiac function after myocardial infarction Circulation 2013 127 213 223 10.1161/CIRCULATIONAHA.112.131110 23224061 PMC3579523

[B6] Li TS Komota T Ohshima M Qin SL Kubo M Ueda K Hamano K TGF-beta induces the differentiation of bone marrow stem cells into immature cardiomyocytes Biochem Biophys Res Commun 2008 366 1074 1080 10.1016/j.bbrc.2007.12.095 18158919

[B7] Lü Y Wang HP Liu B Wu ZG Huo YL Gao CW TGF-β1 induced bone marrow mesenchymal stem cells differentiate in cardiomyocyte-like cells Acta Antomica Sinica 2013 44 49 54

[B8] Behfar A Zingman LV Hodgson DM Rauzier JM Kane GC Terzic A Pucéat M Stem cell differentiation requires a paracrine pathway in the heart FASEB J 2002 16 1558 1566 10.1096/fj.02-0072com 12374778

[B9] Burova E Borodkina A Shatrova A Nikolsky N Sublethal oxidative stress induces the premature senescence of human mesenchymal stem cells derived from endometrium Oxid Med Cell Longev 2013 2013 474931 24062878 10.1155/2013/474931 PMC3767075

[B10] Sarugaser R Hanoun L Keating A Stanford WL Davies JE Human mesenchymal stem cells self-renew and differentiate according to a deterministic hierarchy PLoS One 2009 4 e6498 10.1371/journal.pone.0006498 19652709 PMC2714967

[B11] Senturk S Mumcuoglu M Gursoy-Yuzugullu O Cingoz B Akcali KC Ozturk M Transforming growth factor-beta induces senescence in hepatocellular carcinoma cells and inhibits tumor growth Hepatology 2010 52 966 974 10.1002/hep.23769 20583212

[B12] Wu S Hultguist A Hydbring P Cetinkaya C Oberg F Larsson LG TGF-beta enforces senescence in Myc-transformed hematopoietic tumor cells through induction of Mad1 and repression of Myc activity Exp Cell Res 2009 315 3099 3111 10.1016/j.yexcr.2009.09.009 19766114

[B13] Yu AL Birke K Moriniere J Welge-Lüssen U TGF-{beta}2 induces senescence-associated changes in human trabecular meshwork cells Invest Ophthalmol Vis Sci 2010 51 5718 5723 10.1167/iovs.10-5679 20554622

[B14] Yoon YS Lee JH Hwang SC Choi KS Yoon G TGF beta1 induces prolonged mitochondrial ROS generation through decreased complex IV activity with senescent arrest in Mv1Lu cells Oncogene 2005 24 1895 1903 10.1038/sj.onc.1208262 15688038

[B15] Kong Y Cui H Zhang H Oxidative stress, mitochondrial dysfunction, and aging J Signal Transduct 2012 2012 13 Article ID 646354 10.1155/2012/646354 PMC3184498 21977319

[B16] Velarde MC Flynn JM Day NU Melov S Campisi J Mitochondrial oxidative stress caused Sod2 deficiency promotes cellular senescence and aging phenotypes in the skin Aging (Albany NY) 2012 4 3 12 22278880 10.18632/aging.100423 PMC3292901

[B17] Zhang F Wang C Jing S Ren T Li Y Cao Y Lin J Lectin-like oxidized LDL receptor expresses in mouse bone marrow-derived mesenchymal stem cells and stimulates their proliferation Exp Cell Res 2013 319 1054 1059 10.1016/j.yexcr.2013.01.021 23399833

[B18] Zhang F Wang C Wang H Lu M Li Y Feng H Lin J Yuan Z Wang X Ox-LDL promotes migration and adhesion of bone marrow-derived mesenchymal stem cells via regulation of MCP-1 expression Mediators Inflamm 2013 2013 11 Article ID 691023 10.1155/2013/691023 PMC3730161 23956504

[B19] Zhang W Yang N Shi XM Regulation of mesenchymal stem cell osteogenic differentiation by glucocorticoid-induced leucine zipper (GILZ) J Biol Chem 2008 283 4723 4729 10.1074/jbc.M704147200 18084007

[B20] Lee DH Joo SD Han SB Im J Lee SH Sonn CH Lee KM Isolation and expansion of synovial CD34 (-) CD44 (+) CD90 (+) mesenchymal stem cell: comparison of an enzymatic method and a direct explant technique Connect Tissue Res 2010 52 226 234 21117906 10.3109/03008207.2010.516850

[B21] Maier AB Westendorp RF VAN Heemst D Beta-galactosidase activity as a biomarker of replicative senescence during the course of human fibroblast culture Ann N Y Acad Sci 2007 1100 323 332 10.1196/annals.1395.035 17460195

[B22] Debacq-Chainiaux F Erusalimsky JD Campisi J Toussaint O Protocols to detect senescence-associated beta-galactosidase (SA-betagal) activity, a biomarker of senescent cells in culture and in vivo Nat Protoc 2009 4 1798 1806 10.1038/nprot.2009.191 20010931

[B23] Nelson G Wordsworth J Wang C Jurk D Lawless C Martin-Ruiz C von Zglinicki T Senescent cell bystander effect: senescence-induced senescence Aging Cell 2012 11 345 349 10.1111/j.1474-9726.2012.00795.x 22321662 PMC3488292

[B24] Wang X Khaidakov M Ding Z Dai Y Mercanti F Mehta JL LOX-1 in the maintenance of cytoskeleton and proliferation in senescent cardiac fibroblasts J Mol Cell Cardiol 2013 60 184 190 23648807 10.1016/j.yjmcc.2013.04.024

[B25] Khaidakov M Wang X Mehta JL Potential involvement of LOX-1 in functional consequences of endothelial senescence PLoS One 2011 6 e20964 10.1371/journal.pone.0020964 21698300 PMC3115962

[B26] Swarbrick A Roy E Allen T Bishop JM Id1 cooperates with oncogenic Ras to induce metastatic mammary carcinoma by subversion of the cellular senescence response Proc Natl Acad Sci U S A 2008 105 5402 5407 10.1073/pnas.0801505105 18378907 PMC2291136

[B27] Loeb LA Wallace DC Martin GM The mitochondrial theory of aging and its relationship to reactive oxygen species damage and somatic mtDNA mutations Proc Natl Acad Sci U S A 2005 102 18769 18770 10.1073/pnas.0509776102 16365283 PMC1323222

[B28] Marchi S Giorgi C Suski JM Agnoletto C Bononi A Bonora M De Marchi E Missiroli S Patergnani S Poletti F Rimessi A Duszynski J Wieckowski MR Pinton P Mitochondria-ros crosstalk in the control of cell death and aging J Signal Transduct 2012 2012 17 10.1155/2012/329635 PMC3235816 22175013

[B29] Haorah J Floreani NA Knipe B Persidsky Y Stabilization of superoxide dismutase by acetyl-l-carnitine in human brain endothelium during alcohol exposure: novel protective approach Free Radic Biol Med 2011 51 1601 1609 10.1016/j.freeradbiomed.2011.06.020 21782933 PMC3384514

[B30] Palacios HH Yendluri BB Parvathaneni K Shadlinski VB Obrenovich ME Leszek J Gokhman D Gasiorowski K Bragin V Aliev G Mitochondrial-specific antioxidant as drug treatments for Alzheimer disease CNS Neurol Disord Drug Targets 2011 10 149 162 10.2174/187152711794480474 21222631

[B31] Xing Y Lv A Wang L Yan X The combination of angiotensin II and 5-azacytidine promotes cardiomyocyte differentiation of rat bone marrow mesenchymal stem cells Mol Cell Biochem 2012 360 279 287 10.1007/s11010-011-1067-z 21935612

[B32] Cerrada I Ruiz-Saurí A Carrero R Trigueros C Doronsoro A Sanchez-Puelles JM Diez-Juan A Montero JA Sepúlveda P Hypoxia-inducible factor 1 alpha contributes to cardiac healing in mesenchymal stem cells-mediated cardiac repair Stem Cells Dev 2013 22 501 511 10.1089/scd.2012.0340 22873764

[B33] Zhao JM Zhang MR Ji QY Xing FJ Meng LJ Wang Y The role of slingshot-1 L (SSH1L) in the differentiation of human bone marrow mesenchymal stem cells into cardiomyocyte-like cells Molecules 2012 17 14975 14994 10.3390/molecules171214975 23247370 PMC6268239

[B34] Vidal MA Walker NJ Napoli E Borjesson DL Evaluation of senescence in mesenchymal stem cells isolated from equine bone marrow, adipose tissue, and umbilical cord tissue Stem Cells Dev 2012 21 273 283 10.1089/scd.2010.0589 21410356

[B35] Baker DJ Wijshake T Tchkonia T LeBrasseur NK Childs BG van de Sluis B Kirkland JL van Deursen JM Clearance of p16Ink4a-positive senescent cells delays ageing-associated disorders Nature 2011 479 232 236 10.1038/nature10600 22048312 PMC3468323

[B36] Ahn JH Choi JH Kim JS Lee HJ Lee CH Yoo KY Hwang IK Lee YL Shin HC Won MH Comparison of immunoreactivities in 4-HNE and superoxide dismutases in the cervical and the lumbar spinal cord between adult and aged doges Exp Gerontol 2011 46 703 708 21396438 10.1016/j.exger.2011.03.001

[B37] Tao R Vassilopoulos A Parisiadou L Yan Y Gius D Regulation of MnSOD enzymatic activity by Sirt3 connects the mitochondrial acetylome signaling networks to aging and carcinogenesis Antioxis Redox Signal 2013 20 1646 1654 10.1089/ars.2013.5482 PMC3942696 23886445

[B38] Kasahara E Lin LR Ho YS Reddy VH SOD2 protects against oxidation-induced apoptosis in mouse retinal pigment epithelium: implications for age-related macular degeneration Invest Ophthalmol Vis Sci 2005 46 3426 3434 10.1167/iovs.05-0344 16123448 PMC1237007

